# 

**Published:** 1891-08-22

**Authors:** 


					NNBORNFS
/for INFANTS
ISINGLASS
AND INYALIDS.
No. 256. Yol. X.]
Saturday.
August 22nd, 1891.
[One Penny.1

				

